# Relationship Between the Structure and Immune Activity of Components From the Active Polysaccharides APS-II of Astragali Radix by Enzymolysis of Endo α-1,4-Glucanase

**DOI:** 10.3389/fphar.2022.839635

**Published:** 2022-02-25

**Authors:** Ke Li, Xue-qin Li, Guang-xin Li, Lian-jie Cui, Xue-mei Qin, Zhen-yu Li, Yu-guang Du, Yue-tao Liu, Ai-ping Li, Xing-yun Zhao, Xin-hui Fan

**Affiliations:** ^1^ Modern Research Center for Traditional Chinese Medicine, Shanxi University, Taiyuan, China; ^2^ Institute of Process Engineering, Chinese Academy of Sciences, Beijing, China; ^3^ The Key Laboratory of Chemical Biology and Molecular Engineering of Ministry of Education, Shanxi University, Taiyuan, China; ^4^ Shanxi Key Laboratory of Active Constituents Research and Utilization of TCM, Shanxi University, Taiyuan, China; ^5^ College of Agriculture, Shanxi Agricultural University, Taiyuan, China

**Keywords:** APS-Ⅱ, enzymolysis, structure analysis, immune activity, structure-activity relationship

## Abstract

Astragali Radix polysaccharides (APSs) have a wide range of biological activities. Our preliminary experiment showed that APS-Ⅱ (10 kDa) was the main immunologically active component of APSs. However, the characteristic structure related to activity of APS-Ⅱ needs further verification and clarification. In this study, APS-II was degraded by endo α-1,4-glucosidase. The degraded products with different degrees of polymerization [1–3 (P1), 3–6 (P2), 7–14 (P3), and 10–18 (P4)] were obtained using a polyacrylamide gel chromatography column. The structural features of the different products were characterized by HPGPC, monosaccharide composition, Fourier transform infrared spectrum, GC–MS, nuclear magnetic resonance, and UPLC-ESI-QTOF-MS analysis. Specific immune and non-specific immune cell tests were used to identify the most immunogenic fractions of the products. The backbone of P4 was speculated to be α-D-1,4-linked glucans and rich in C2 (25.34%) and C6 (34.54%) branches. Immune screening experiments indicated that the activity of P4 was better than that of APS-II and the other three components. In this research, the relationship between the structure of APS-Ⅱ and the immune activity from the degradation level of polysaccharides was studied, laying a foundation for the quality control and product development of APSs.

## Introduction

*Astragalus mongholicus* Bunge is the dried root of the perennial legume *Astragalus membranaceus* (Fisch.) Bge. var. mongholicus (Bge.) Hsiao or *Astragalus membranaceus* (Fisch.) Bge. It is widely used in traditional Chinese medicine prescriptions. Polysaccharides are one of the main active ingredients of AR. Various pharmacological studies have proven that Astragali Radix polysaccharides (APSs) have immunomodulatory (Liu AJ. et al., 2017; Wan et al., 2022), antitumor (Li W. et al., 2020), antidiabetic (Zhang et al., 2019), and antiviral effects (Zheng et al., 2020). Thus, they have great potential and broad prospects. These pharmacological activities are based on immune regulation, including specific and nonspecific immunity (Lobner, et al., 2008; Li et al., 2019). In terms of innate immunity, APSs can stimulate the proliferation of immune cells, such as phagocytes, natural killer cells, and dendritic cells. In terms of adaptive immunity, APSs exert immune activity by activating T and B lymphocytes to directly kill target cells or promoting the production of various cytokines. APSs have a wide molecular weight distribution and complex branch structure (Jin et al., 2014). Due to the limitations of the current separation and structural analysis technology of polysaccharides, the fine structure cannot be accurately determined, which limits the in-depth study of the structure–activity relationship of APSs.

Current research on APSs mainly focuses on the study of the structures and immune activities of total polysaccharides or on a certain component of APSs. Systematic structure study and immunoactivity screening of APSs with different molecular weights have not been conducted. In the early stage, the immune activity *in vitro* of APSs with different molecular weights was screened. We found that APS-Ⅱ (molecular weight, 10 kDa) showed strong immunoenhancing activity in specific and non-specific immunity. However, *in-vivo* experimental screening of cyclophosphamide immunosuppressive mice verified that APS-Ⅱ was the component with the strongest immune-promoting activity of APSs (Li et al., 2020a). The results of high performance gel permeation chromatography (HPGPC) showed a single symmetrical peak, indicating that APS-Ⅱ were well purified and homogeneous polysaccharide components (Li et al., 2020a). The structure of APS-Ⅱ were clarified by monosaccharide composition, linkage analysis, Fourier transform infrared spectroscopy (FT–IR), nuclear magnetic resonance (NMR). In the monosaccharide composition of APS-Ⅱ, the most abundant was Glu, followed by Ara and Gal. APS-II monosaccharide residue was linked to →5)-α-L-Ara-(1→, →4)-α-D-Glu-(1→, →3,4,6)-β-D-Glu-(1→, etc. Although the preliminary structural analysis of APS-Ⅱ has been performed, the fine structure and the characteristic structure related to activity still needs to be further verified and explained.

At present, the research on protein, nucleic acid, and other biological macromolecules is relatively mature, while that on polysaccharides is only the tip of the iceberg (Li and Huang, 2021). In the study of protein structure, the “bottom-up” method is often used to degrade the protein into peptides or amino acids for characterization and analysis (Moreno-Gordaliza et al., 2010; 2012). Compared with the degree of polymerization of polysaccharides with large molecular weights and complex structures, that of oligosaccharides is reduced, which could explain the primary structure of polysaccharides more clearly through high resolution mass spectrometry and NMR. Oligosaccharides have better water solubility and are easier to enter the organism through multiple cell membrane barriers to play a role. Using a “bottom-up” research strategy, we degrade polysaccharides into oligosaccharides, and explain their primary structure and immune activity characteristics at the oligosaccharide level (Liang et al., 2014). At present, the common methods for degrading polysaccharides include chemical degradation and enzymatic degradation. Compared with chemical degradation, enzymolysis could cut specific glycosidic bonds. The enzymolysis reaction is easy to control, and that is reported to be gentler and more efficient (Yan et al., 2021; Albuquerque et al., 2022), hence an ideal degradation method. In this study, to further verify the key structural characteristics of APS-II with immunological activity, APS-II was degraded into a mixture of oligosaccharides by endo α-1,4-glucosidase. The polyacrylamide gel column was used to separate and prepare sugar components with different degrees of polymerization. The structural differences of the four fractions were clarified by monosaccharide composition, linkage analysis, Fourier transform infrared spectroscopy (FT–IR), nuclear magnetic resonance (NMR), and mass spectrometry analysis. Cellular immune activity experiments for nonspecific and specific immunoactivity screening were performed to identify the most immunologically active fragments of APS-II enzymatic digestion components. This study aimed to clarify the relationship between the key structure and the immune activity of APS-II by comparing the structure and activity of saccharides with different degrees of polymerization after enzymolysis.

## Materials and Methods

### Materials

Wild-simulated AR was obtained from Shanxi Hunyuan and identified by Professor Qin Xuemei of Shanxi University as the dry root of *Astragalus mongholicus* Bunge (harvested in 2017, growth period of 5 years). The medicinal material samples were kept in the sample bank of the Modern Research Center of Traditional Chinese Medicine of Shanxi University. Polyacrylamide gel Bio-Gel-P-2 was purchased from Bio-Rad (USA). Endo α-D-1,4-glucanohydrolase was obtained from Beijing Soleibao Technology Co., Ltd. Standard oligosaccharide samples were obtained from Glycarbo (Japan), and standard monosaccharide samples were purchased from Shanxi Jiujiu Trading Co., Ltd. The ELISA kit for mouse immunoglobulin G and cell counting kit (CCK-8) were acquired from Soleibao Technology Co., Ltd. Mouse monocyte macrophage Raw 264.7 and mouse lymphoma cells (YAC-1) were obtained from ATCC (American Type Culture Collection). Male BALB/c mice were purchased from Weitong Lihua Laboratory Animal Technology Co., Ltd. Phosphate buffered saline solution, lipopolysaccharide, trypsin, concanavalin A, and neutral red were purchased from Soleibao Technology Co., Ltd. Fetal bovine serum, DMEM high-glucose media and RPMI 1640 media were obtained from Soleibao Technology Co., Ltd.

### Preparation of Enzymolysis Components From APS-II (APOS)

APS-II was prepared in accordance with the previous method (Li et al., 2020a). The single factor and orthogonal test of enzymolysis of APS-II by endo α-1,4-glucanase results showed that the best enzyme degradation conditions were 0.5 U/ml (enzyme concentration), 60°C (enzymolysis temperature), and 90 min (enzymolysis time). Approximately 2 ml of polysaccharide solution (1 mg/ml) was mixed with 2 ml of endo α-1,4-glucanase solution (0.5 U/ml) and degraded at 60°C for 90 min. After enzymolysis, the solution was inactivated by boiling water for 10 min. The supernatant was collected and freeze dried for the preparation of APOS.

The APOS solution was separated on a polyacrylamide gel (Bio-Gel-P-2) chromatography column to obtain sugar components with different degrees of polymerization. First, Bio-Gel-P-2 solution was loaded onto a glass column (1.5 cm × 70 cm) to 60 cm, and the solution was sequentially eluted with deionized water at a flow rate of 8.8 ml/h. Fractions with different degrees of polymerization were obtained due to the elution order of the peak. Sixty tubes were collected, the volume of the collected liquid in each tube was 1.5 ml and high-performance liquid chromatography (HPLC) analysis was performed for every five tubes. Tubes at 1–27 were collected and named P4, tubes at 28–34 were collected and named P3, tubes at 35–42 were collected and named P2, and tubes at 43–60 were collected and named P1. Afterwards, lyophilization was carried out to obtain four saccharide fractions with different degrees of polymerization (P1–P4). The chromatographic column was Huapu X-Amide (250 mm × 4.6 mm, 5 μm), the detector was ELSD, and the mobile phase contained A (pure water) and B (acetonitrile). Gradient elution was performed with the following parameters: 0–55 min, 15%–50% A, and 85–50% B. The degree of polymerization of the saccharides in the enzymolysis product was determined using standard dextran.

### Monosaccharide Composition Analysis and Linkage Analysis

The PMP derivatization method (Li et al., 2020b) was used in determining the monosaccharide composition. Briefly, APOS and saccharide components with different degrees of polymerization (P1–P4) were hydrolyzed with 2 M TFA at 120°C for 2 h. The released monosaccharides were derived with PMP. In addition, 2695 HPLC equipped with a Venusil XBP C18 column (4.6 mm × 250 mm, 5 μm) and 2489 UV detector were used.

The method of linkage analysis through methylation was obtained from the literature (Bai et al., 2020; Li et al., 2020c). Briefly, the dried APOS and saccharide components with different degrees of polymerization (P1–P4) (5 mg) was completely dissolved in anhydrous DMSO (2 ml), and reacted with anhydrous NaH (50 mg) and methyliodide (1 ml, three times). The methylated polysaccharides were extracted by chloroform, and complete methylation was confirmed by FT-IR analysis. After that, the methylated products were hydrolyzed with 2 M TFA at 120°C for 90 min, reduced by 2% NaBD_4_, and finally acetylated with acetic anhydride and 1-methylimidazole at 25°C for 10 min. The obtained alditol acetates were analyzed by GC–MS with a DB-5MS capillary column. The oven temperature was as follows: gradual increase from 100 to 180°C at 5°C/min, with a hold time of 1 min; to 190°C at 1°C/min, with a hold time of 2 min; to 220°C at 30°C/min, with a hold time of 2 min; to 230°C at 1°C/min, with a hold time of 2 min; and to 280°C at 20°C/min, with a hold time of 10 min (Prozil et al., 2012).

### FT–IR analysis of APS-II , APOS and P1–P4

Approximately 4 mg of APS-II, APOS and P1–P4 were mixed with KBr powder and pressed into 1 mm granules. A Fourier transform infrared spectrometer (Nicolet IS50, Thermo Scientific, USA) was used in scanning within a wavelength range of 4000–400 cm^−1^ (Dong et al., 2020).

### NMR Spectroscopy Analysis of APOS

Thirty milligrams of dried APOS was proton exchanged in 0.5 ml of deuterium oxide (D_2_O, 99.9%) containing trimethylsilylpropionate (TSP) (δ H and δ C = 0.00) as an internal standard. (Wu et al., 2020). ^1^H-NMR, ^13^C-NMR, heteronuclear singular quantum correlation (HSQC), and ^1^H detected heteronuclear multiple bond correlation (HMBC) were recorded with a 600 MHz spectrometer (AVANCE Ⅲ HD 600 MHz, Bruker, Germany) at 25°C. Chemical shifts were given in ppm.

### Mass Spectrometry Analysis of APOS

The APOS was analyzed with a UPLC–ESI–QTOF–MS system equipped with an Acquity UPLC BEH amide column (10 mm × 2.1 mm, 1.7 μm) at a flow rate of 0.3 ml/min. The column temperature was set at 40°C, and the sample room temperature was 4°C. Mobile phase A consisted of 0.1% formic acid in water, and mobile phase B consisted of acetonitrile. The optimized UPLC conditions were as follows: 0–2 min, 65% B; 2–6 min, 65%–60% B; 6–7 min, 60%–56% B; 7–8 min, 56% B; 8–9 min, 56%–52% B; 9–10 min, 52%–56% B; 10–11 min, 56% B; 11–12 min, 56%–65% B; and 12–13 min, 65% B. ESI–MS experiment was carried out in negative ion mode at 100°C.

### Effects of P1–P4 on the Phagocytic Activity of Raw 264.7 Cells

Phagocytic activity was evaluated as previously reported, with slight modification (Li et al., 2020a). Raw 264.7 cells were treated with APS-II, APOS, and P1–P4 with different concentrations (10, 20, 30, 50, 70, and 100 μg/ml). APS-II was used as a positive control, and a blank control group was established. After culturing for 24 h, neutral red dye (0.075%) was added, and incubation was performed for another 4 h. Then, the supernatant was removed, and the cells were washed with PBS twice. Subsequently, 200 μl of cell lysate (ethanol:acetic acid = 1:1) was added and incubated overnight. The absorbance was measured at 540 nm.

### Effects of P1–P4 on the Killing Activity of NK Cells

Splenocytes (effector cells) and YAC cells (target cells) were added to a 96-well plate, and the effective-to-target ratio was 50:1. The control group had only target or effector cells. The experimental group was added with APS-II, APOS, and P1–P4 at different concentrations (10, 25, 50, 100, 200, and 400 μg/ml). After 12 h of incubation, CCK-8 experiment was performed in accordance with the kit instructions. NK cell cytotoxicity = (1 – [ODS − ODE]/ODT) × 100% (where ODS is the absorbance value of the sample group; ODE is the absorbance value of the effector cell and ODT is the absorbance value of the target cell of the control group).

### Effects of P1–P4 on Lymphocyte Proliferation

Mouse spleen cell suspension was prepared (Yao et al., 2018; Ji et al., 2021) and seeded into a 96-well plate at a concentration of 1 × 10^7^ cells/ml. APS-II, APOS, and P1–P4 solutions with different concentrations were added. ConA (final concentration of 50 μg/ml) and LPS (final concentration of 10 μg/ml) were employed to stimulate splenic T lymphocytes and B lymphocytes in each group. Then, they were added successively before the solution was incubated for 48 h, and the proliferative activities were evaluated by CCK-8 kit.

### Effects of P1–P4 on the Secretion of IgG From Splenic Lymphocytes

Mouse splenic lymphocytes were seeded into 96-well plate at a concentration of 5 × 10^5^ cells/ml. Different concentrations (10, 25, 50, 100, 200, and 400 μg/ml) of APS-II, APOS, and P1–P4 were added to each well. After culturing for 48 h and centrifugation, the supernatant was collected (Xu et al., 2019). The IgG content was detected through ELISA.

### Statistical Methods

Data were processed with Prism 7.0 software, and the difference between the groups was analyzed using *t*-test. The experimental data were expressed as ± s, and the test level was α = 0.05. *p* < 0.05 indicated statistical significance.

## Results and Discussion

### Separation and Preparation of P1–P4

**FIGURE 1:**
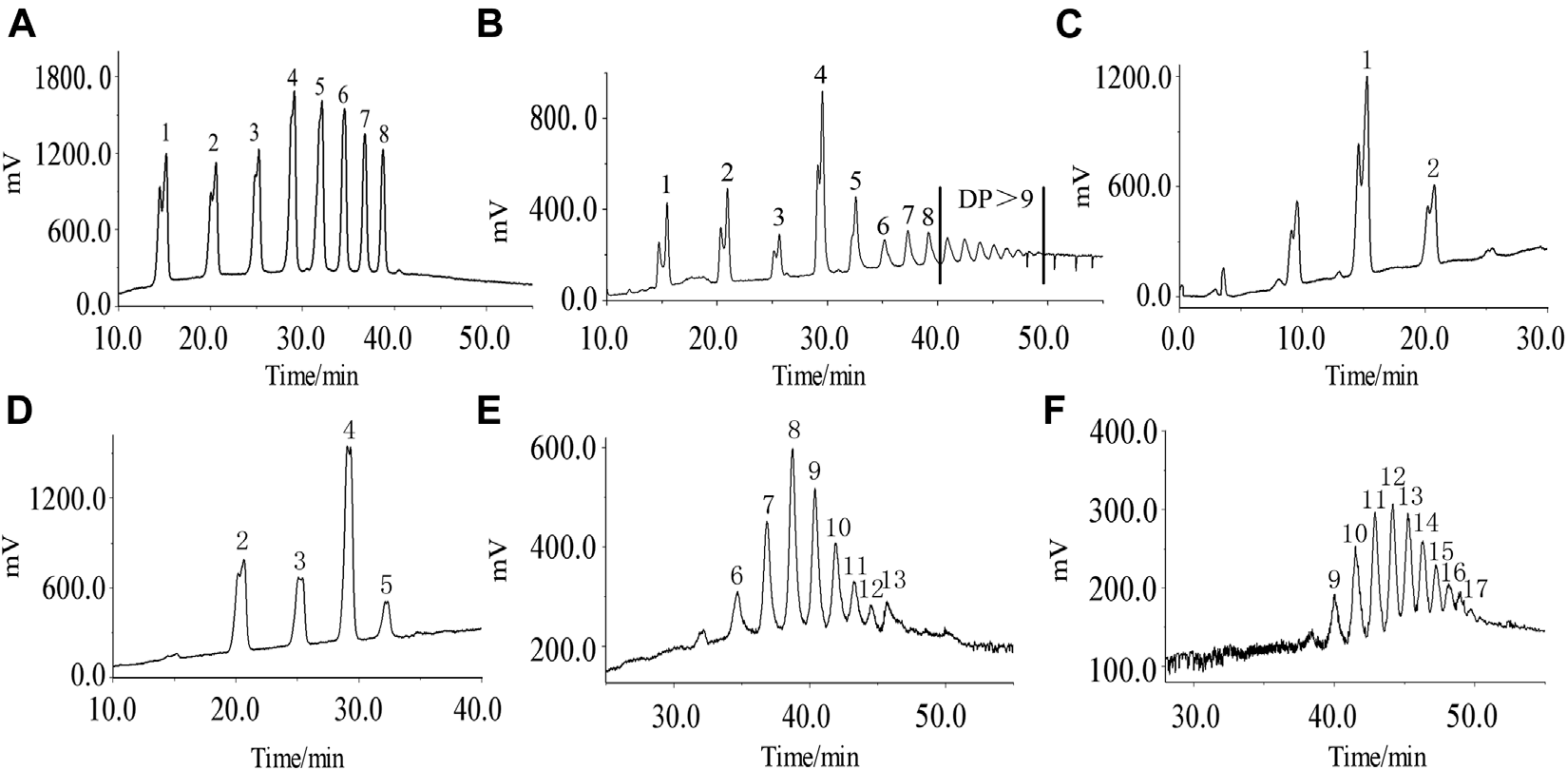
HPLC chromatogram of standard oligosaccharide samples **(A)**, APOS **(B)**, P1 **(C)**, P2 **(D)**, P3 **(E)**, and P4 **(F)**. 1–17: chromatographic peak of dextran with degree of polymerization of 2–18.

The degree of polymerization of the saccharide components after the enzymatic degradation of APS-II could be inferred in accordance with the retention time of the standard dextran two to nine sugars (Figure 1A). Saccharides with a degree of polymerization of 1–18 after enzymolysis could be found (Figure 1B). APOS was separated by Bio-Gel-P-2 gel column to four saccharide fractions, with degrees of polymerization 1–3 (P1), 3–6 (P2), 7–14 (P3), and 10–18 (P4), as shown in Figures 1C–F. This finding provided a material basis for structural analysis and immune activity research.

### Analysis of Monosaccharide Composition of P1–P4

**FIGURE 2:**
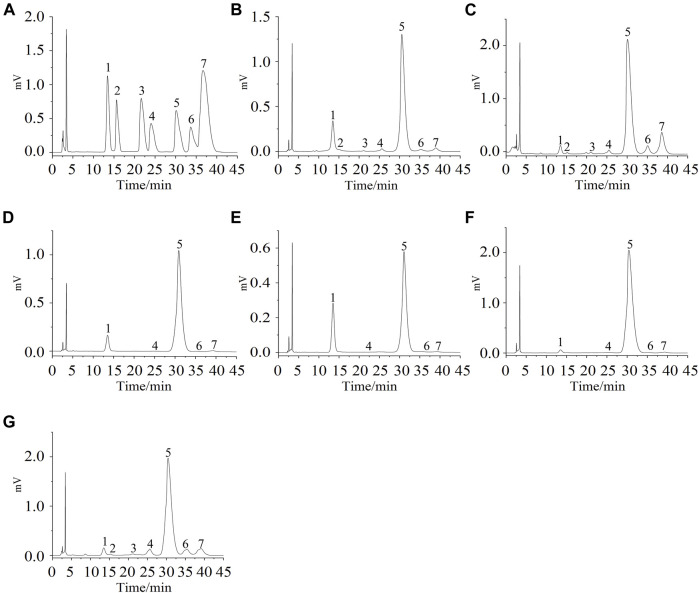
Mixture monosaccharide standard of HPLC–UV chromatogram **(A)**; **(B**–**G)** indicate the HPLC–UV chromatogram of APS-II, APOS, P1, P2, P3, and P4.1: PMP, 2: glucuronic acid, 3: rhamnose, 4: galacturonic acid, 5: glucose, 6: galactose, 7: arabinose.

The composition of monosaccharides was determined by HPLC–PMP–UV. The results are shown in Figure 2 and Table 1. APS-II was mainly composed of glucuronic acid, rhamnose, galacturonic acid, glucose, galactose, and arabinose in ratios of 0.32, 0.56, 1.70, 79.85, 3.86, and 13.71%, respectively. APOS was mainly composed of glucuronic acid, rhamnose, galacturonic acid, glucose, galactose, and arabinose in ratios of 0.25, 0.30, 1.58, 93.70, 1.40, and 2.76%, respectively. P1 was mainly composed of galacturonic acid, glucose, galactose, and arabinose in ratios of 0.33, 98.18, 0.20, and 1.29%, respectively. P2 was primarily composed of galacturonic acid, glucose, galactose, and arabinose in ratios of 0.48, 98.38, 0.56, and 0.58%, respectively. P3 was mainly composed of galacturonic acid, glucose, galactose, and arabinose in ratios of 0.29, 98.88, 0.02, and 0.81%, respectively, while P4 consisted of glucuronic acid, rhamnose, galacturonic acid, glucose, galactose, and arabinose in ratios of 0.24, 0.63, 3.86, 85.67, 3.88, and 5.72%, respectively. In summary, the main component of P1, P2, and P3 was glucose, followed by arabinose, galacturonic acid, and galactose. However, the main components in P4 and APOS contained glucuronic acid, rhamnose, galacturonic acid, glucose, galactose and arabinose, and the proportion of each monosaccharide was different. Notably, glucose may be the backbone of the main chain of P1–P4.

**TABLE 1:** Results of monosaccharide composition analysis of different saccharide components.

Sugar component	Monosaccharide composition (molar ratio, %)					
	Glucuronic acid	Rhamnose	Galacturonic acid	Glucose	Galactose	Arabinose
APS-II	0.32	0.56	1.70	79.85	3.86	13.71
APOS	0.25	0.30	1.58	93.70	1.40	2.76
P1	—	—	0.33	98.18	0.20	1.29
P2	—	—	0.48	98.38	0.56	0.58
P3	—	—	0.29	98.88	0.02	0.81
P4	0.24	0.63	3.86	85.67	3.88	5.72

Comparison of APS-II, APOS, and P1–P4 by FT–IR.

### Comparison of APS-II, APOS, and P1–P4 by FT–IR

**FIGURE 3:**
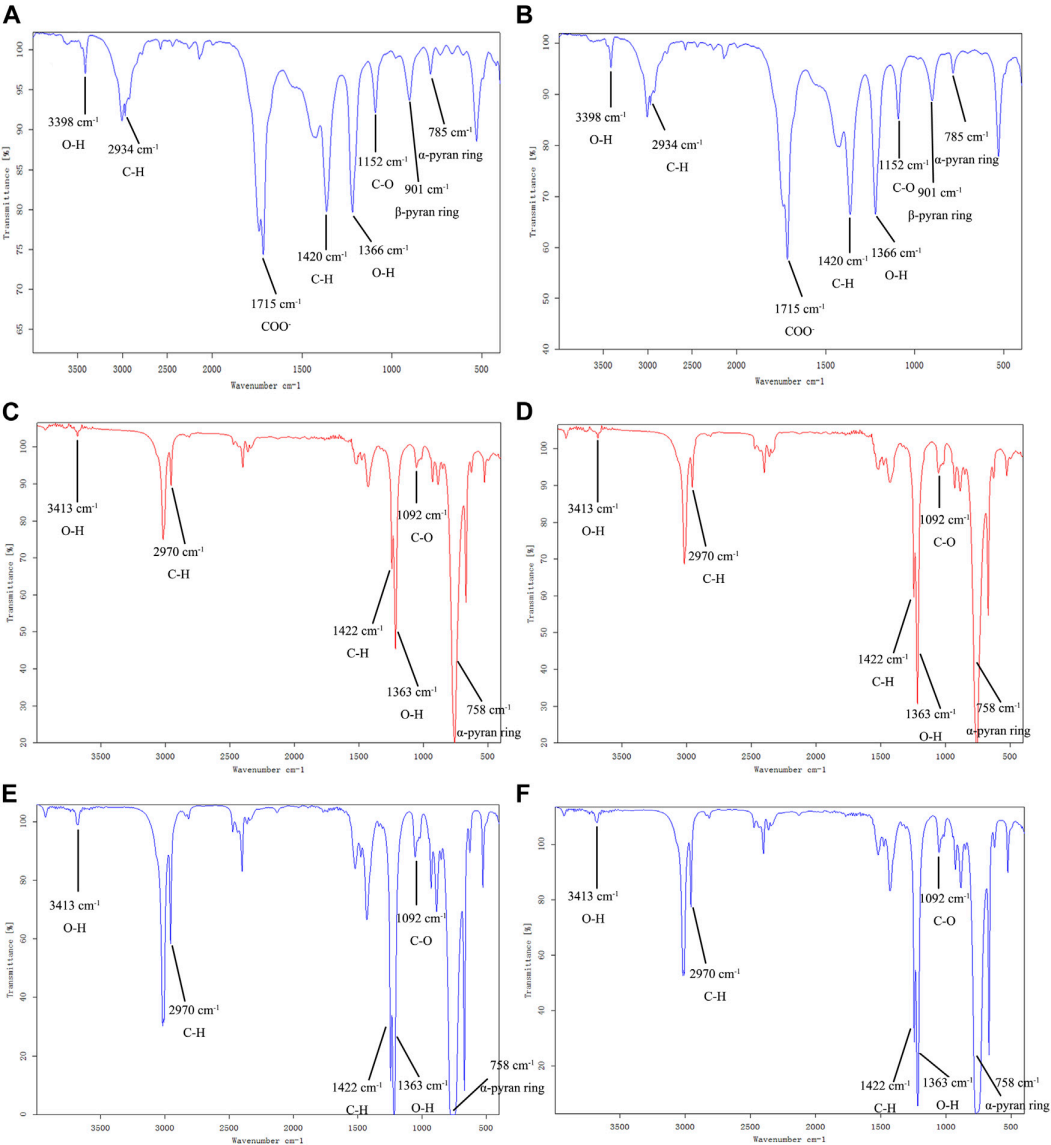
FT-IR spectra of APS-II **(A)**, APOS **(B)**, P1 **(C)**, P2 **(D)**, P3 **(E)** and P4 **(F)**.

The FT-IR spectrum of APS-II and APOS were similar (Figures 3A,B). The broad absorption around 3398 cm^−1^ and the sharp absorption at 2934 cm^−1^ were assigned to the O–H stretching vibration and C–H stretching vibration. The absorption band at 1715 cm^−1^ indicated the COO^−^ of uronic acid. The absorption peaks at 1420 cm^−1^ and 1366 cm^−1^ were attributed to the bending vibrations of C–H and O–H, respectively (Liu J. et al., 2021). The absorption peak at 1152 cm^−1^ could be attributed to the C–O stretching vibration of the pyranose ring, which is a typical IR signal of glucan (Wu T. et al., 2021). The peak at 785 cm^−1^ was the symmetric stretching vibration of the α-pyran ring; the peak at 901 cm^−1^ could be attributed to the β-pyran ring.

The FT-IR spectrum (Figures 3C–F) of P1, P2, P3 and P4 displayed the broad and intense stretching peak at 3413 cm^−1^ which related to stretching vibration of the hydroxyl groups. The band at 2970 cm^−1^ was a characteristic of C-H stretching vibration. The absorption peaks at 1422 cm^−1^ and 1363 cm^−1^ were assigned to the bending vibrations of C–H and O–H, respectively. The absorption peak at 1092 cm^−1^ was assigned with C-O stretching vibration. The absorption at 758 cm^−1^ was attributed to α-pyran ring. It can be seen from the infrared spectrum that P1, P2, P3 and P4 have obvious characteristics of carbohydrate compounds. The distribution of absorption peaks in the FT-IR spectrum of P1, P2, P3 and P4 was similar to that of APS-II and APOS, but the peak intensities were different, which may be related to the proportion of functional groups in their structures.

### Methylation Analysis of P1–P4

Methylation analysis is the most common method to determine how monosaccharide units are connected in the sugar chain of oligosaccharides and polysaccharides. The total ion chromatograms of different saccharide components were shown in Supplementary Figure S1. The mass spectrum fragment ion peaks of different saccharide components in the GC-MS spectrum were compared with the mass spectra in the database (US CCRC data https://www.ccrc.uga.edu/specdb/ms/pmaa/pframe.html) and literature reference (Kochetkov and Chizhov, 1965; Sassaki et al., 2005; Sims et al., 2018; Xia et al., 2020). Peak areas were used in calculating the molar ratio of each sugar residue. Table 2 summarizes the connection mode of APOS, P1, P2, P3, and P4 sugar residues. The results of methylation analysis revealed that the largest residue in the P1 structure was →4)-Glcp-(1→ (59.52%) and Glcp-(1→ (18.45%). The second largest residues in the structure were →6)-Glcp-(1→ (8.33%), →2,4,6)-Glcp-(1→ (4.76%) and →2,3,4)-Glcp-(1→ (5.59%), followed by the number of →3,4,6-Glcp-(1→ (2.98%). P2 was similar to P1. Compared with the linkages in P1 and P2, the number of Glcp-(1→ (13.82%), →4)-Glcp-(1→(46.08%), →6)-Glcp-(1→ (5.07%), →2,4,6)-Glcp-(1→ (3.22%), →2,3,4)-Glcp-(1→ (3.69%) decreased in P3. In addition, Araf-(1→, Galp-(1→, →5)-Araf-(1→, →2,4,6)-Galp-(1→, →2,4)-Glcp-(1→ and →4,6)-Glcp-(1→ appeared. In the saccharide fraction with P4, the main glycosidic linkages were →2,4-Glcp-(1→ (25.34%) and →4,6-Glcp-(1→ (34.54%). The number of →4)-Glcp-(1→ (3.50%) and Glcp-(1→ (3.01%) decreased. Glucose with a high degree of branching was the main component. Similarly, the following 14 components Araf-(1→, Galp-(1→, →5)-Araf-(1→, Glcp-(1→, →4,6)-Glcp-(1→, →2,4)-Glcp-(1→, →2,3)-Glcp-(1→, →4)-Glcp-(1→, →6)-Glcp-(1→, →2,3,4)-Galp-(1→, →2,3,4)-Glcp-(1→, →2,4,6)-Galp-(1→, →2,4,6)-Glcp-(1→, →3,4,6)-Glcp-(1→ were found in APOS at molar ratios of 3.92: 3.14: 8.64: 14.14: 9.69: 2.88: 6.28: 26.18: 2.09: 1.31: 1.57: 9.42: 7.85: 2.88, respectively.

**TABLE 2:** Results of methylation analysis of different saccharide components.

O-methylated alditol acetates	Linkage type	Mass fragment, m/z	Molar ratio %				
			P1	P2	P3	P4	APOS
2,3,5-Me_3_-Ara	Araf-(1→	75, 87, 88, 101, 111, 118, 127, 143, 145, 234	—	—	2.30	8.71	3.92
2,3,4,6-Me_4_-Gal	Galp-(1→	71, 87, 101, 111, 118, 127, 129, 145, 157, 205	—	—	2.76	6.65	3.14
3,5-Me_2_-Ara	→5)-Araf-(1→	59, 71, 87, 102, 118, 129, 189	—	—	0.92	1.05	8.64
2,3,4,6-Me_4_-Glc	Glcp-(1→	71, 87, 101, 102, 118, 129, 145, 161, 162, 205	18.45	21.23	13.82	3.01	14.14
2,3-Me_2_-Glc	→4,6)-Glcp (1→	73, 85, 87, 111, 127, 142, 171, 201, 233	—	—	5.53	34.54	9.69
2,6-Me_2_-Glc	→2,4)-Glcp (1→	71, 87, 99, 113, 130, 140, 173, 190, 233	—	—	6.45	25.34	2.88
4,6-Me_2_-Glc	→2,3)-Glcp (1→	45, 85, 87, 101, 115, 129, 161, 202, 232	—	—	—	4.52	6.28
2,3,6-Me_3_-Glc	→4)-Glcp-(1→	71, 87, 99, 117, 118, 129, 131, 141, 162, 173, 233	59.52	55.87	46.08	3.50	26.18
2,3,4-Me_3_-Glc	→6)-Glcp-(1→	71, 88, 99, 102, 118, 131, 173, 191, 233	8.33	6.14	5.07	—	2.09
6-Me-Gal	→2,3,4)-Galp (1→	45, 87, 99, 115, 129, 143, 157, 171, 185, 201, 231, 261	—	—	—	3.68	1.31
6-Me-Glc	→2,3,4)-Glcp (1→	87, 99, 115, 129, 157, 171, 185, 218, 231, 261	5.95	8.38	3.69	2.63	1.57
3-Me-Gal	→2,4,6)-Galp (1→	73, 85, 87, 99, 127, 130, 142, 159, 190, 201, 261	—	—	5.07	3.22	9.42
3-Me-Glc	→2,4,6)-Glcp (1→	73, 87, 99, 115, 130, 142, 159, 171, 190, 261	4.76	5.59	3.22	1.89	7.85
2-Me-Glc	→3,4,6)-Glcp (1→	73, 87, 97, 118, 139, 160, 171, 187, 202, 213, 231, 259	2.98	2.79	5.07	1.26	2.88

### NMR Spectroscopy Analysis of APOS

**FIGURE 4:**
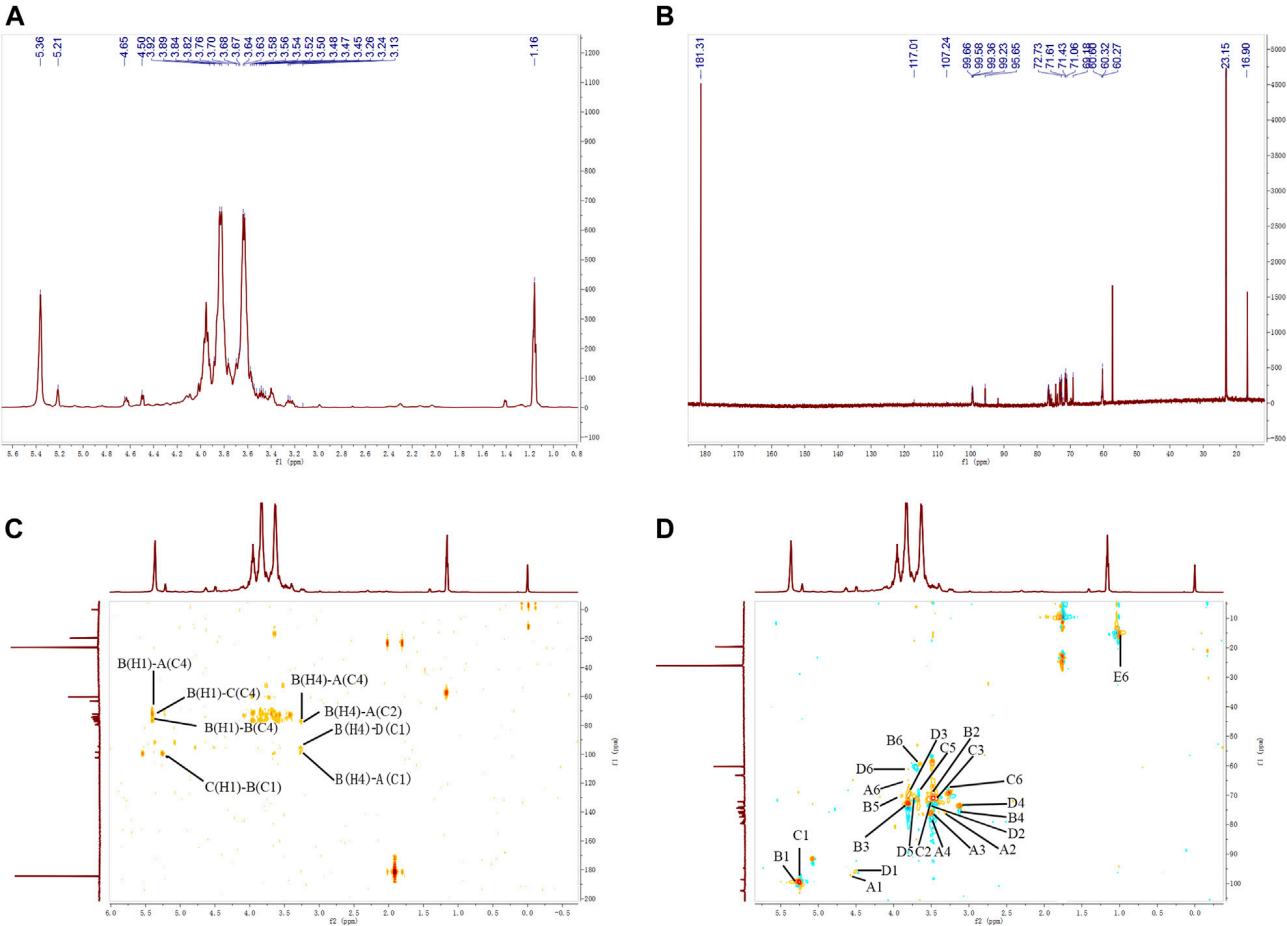
^1^H–NMR **(A)**, ^13^C–NMR **(B)**, HMBC **(C)**, and HSQC **(D)** spectra of APOS.

The ^1^H–NMR, ^13^C–NMR, ^1^H–^13^C HSQC, and ^1^H–^13^C HMBC spectra of APOS are shown in Figure 4. The chemical shift of the polysaccharide anomeric carbon at 4–6 ppm was observed in the hydrogen spectrum, and the polysaccharide anomeric carbon in the carbon spectrum appeared at 90–110 ppm. In the hydrogen spectrum, the residues with anomeric proton chemical shifts exceeding 4.7 ppm were in α-configuration, and the chemical shift below 4.7 ppm indicated the existence of β-configuration. In the carbon spectrum, the α-configuration was in the range of 95–101 ppm, and the β-configuration was in the range of 101–105 ppm (Synytsya and Novak, 2014; Patra et al., 2021; Zhang et al., 2021).

As shown in Figure 4, the ^13^C NMR spectrum of APOS showed a series of anomeric resonance at δ 99.36, 99.65, 99.66, and 99.23 ppm, which corresponded to the anomeric carbons of →2,4)-*α*-D-Glc*p*-(1→ (Residue **A**), →4)-*α*-D-Glc*p*-(1→ (Residue **B**), →4,6)-*α*-D-Glc*p*-(1→ (Residue **C**) and *α*-D-Glc*p*-(1→ (Residue **D**), respectively. Their correlated anomeric proton signals were assigned at δ 5.36, 5.36, 5.21, and 4.65 ppm by HSQC. The signals at δ 181.31 ppm in the region for the resonances of the carboxyl groups corresponded to the C-6 regions of the unesterified galacturonic acid units (Yao et al., 2021). The signal at δ 16.90 ppm confirmed the presence of rhamnosyl residues (Wu M. et al., 2021). The chemical shifts of the sugar residues were designated and shown in Table 3.

**TABLE 3:** Signals of chemical shifts in ^1^H and ^13^C NMR spectra of APOS.

Residues	Chemical shifts (ppm)					
	C1/H1	C2/H2	C3/H3	C4/H4	C5/H5	C6/H6
Residue A, →2,4)-*α*-D-Glc*p*-(1→	99.36	76.77	75.63	76.48	71.23	60.27
	5.36	3.48	3.63	3.52	3.67	3.92
Residue B, →4)-*α*-D-Glc*p*-(1→	99.65	71.54	73.20	76.89	71.61	60.32
	5.36	3.56	3.76	3.24	3.89	3.70
Residue C, →4,6)-*α*-D-Glc*p*-(1→	99.66	71.43	73.07	76.68	72.73	69.18
	5.21	3.45	3.47	3.50	3.64	3.58
Residue D, *α*-D-Glc*p*-(1→	99.23	72.60	71.35	71.06	71.17	60.60
	4.65	3.54	3.82	3.26	3.68	3.84
Residue E, *α*-L-Rha	99.58	72.14	73.23	73.17	72.55	16.90
	—	—	—	—	—	1.16

The heteronuclear ^1^H-^13^C HSQC could reflect the directly connected C-H coupling relationship, and the remote heteronuclear ^1^H-^13^C HMBC could provide glycosidic bond connection. The chemical shifts at δ 3.92/60.27, 3.67/71.23, 3.52/76.48, 3.63/75.63, 3.48/76.77, and 5.36/99.36 ppm on the HSQC spectrum (Figure 4C) corresponded to the H-6/C-6, H-5/C-5, H-4/C-4, H-3/C-3, H-2/C-2, and H-1/C-1 of residue **A**, respectively. The chemical shifts at δ 3.70/60.32, 3.89/71.61, 3.24/76.89, 3.76/73.20, and 3.56/71.54 ppm corresponded to the H-6/C-6, H-5/C-5, H-4/C-4, H-3/C-3, H-2/C-2, and H-1/C-1 of residue **B**, respectively. The chemical shifts at δ 3.58/69.18, 3.64/72.73, 3.50/76.68, 3.47/73.07, 3.45/71.43, and 5.21/99.66 corresponded to the H-6/C-6, H-5/C-5, H-4/C-4, H-3/C-3, H-2/C-2, and H-1/C-1 of residue **C**, respectively. The chemical shifts at δ 3.84/60.60, 3.68/71.17, 3.26/71.06, 3.82/71.35, 3.54/72.60, and 4.65/99.23 ppm corresponded to the H-6/C-6, H-5/C-5, H-4/C-4, H-3/C-3, H-2/C-2, and H-1/C-1 of residue **D**, respectively. The chemical shifts at δ 1.16/16.90 ppm corresponded to the H-6/C-6 of residue **E**. The sequence and linkage sites among residues were further confirmed by analyzing the signals of the ^1^H-^13^C HMBC spectrum of APOS. The cross peaks were assigned as follows: H-1 (5.36 ppm) for residue **B** and C-4 (76.48 ppm) for residue **A** (BH1/AC4), suggesting that the O-1 of residue **B** was linked to the C-4 of residue **A**. Similarly, the cross peaks at δ 5.36/76.68 (BH1/CC4), 5.21/99.65 (CH1/BC1), 3.24/76.48 (BH4/AC4), 3.24/76.77 (BH4/AC2), 3.24/99.23 (BH4/DC1), and 3.24/99.36 (BH4/AC1) ppm suggested that the O-1 of residue **B** was linked to the C-4 of residue **C**, the O-1 of residue **C** was linked to the C-1 of residue **B**, the O-4 of residue **B** was linked to the C-4 of residue **A**, the O-4 of residue **B** was linked to the C-2 of residue **A**, the O-4 of residue **B** was linked to the C-1 of residue **D**, and the O-4 of residue **B** was linked to the C-1 of residue **A**, respectively. In addition, 5.36/76.89 ppm (BH1/BC4) suggested that the O-1 of residue **B** was linked to the C-4 of residue **B** in APOS (Xia et al., 2019; Huo et al., 2020; Xia et al., 2020; Patra et al., 2021; Yao et al., 2021).

### UPLC-ESI-QTOF-MS Analysis of APOS

**FIGURE 5:**
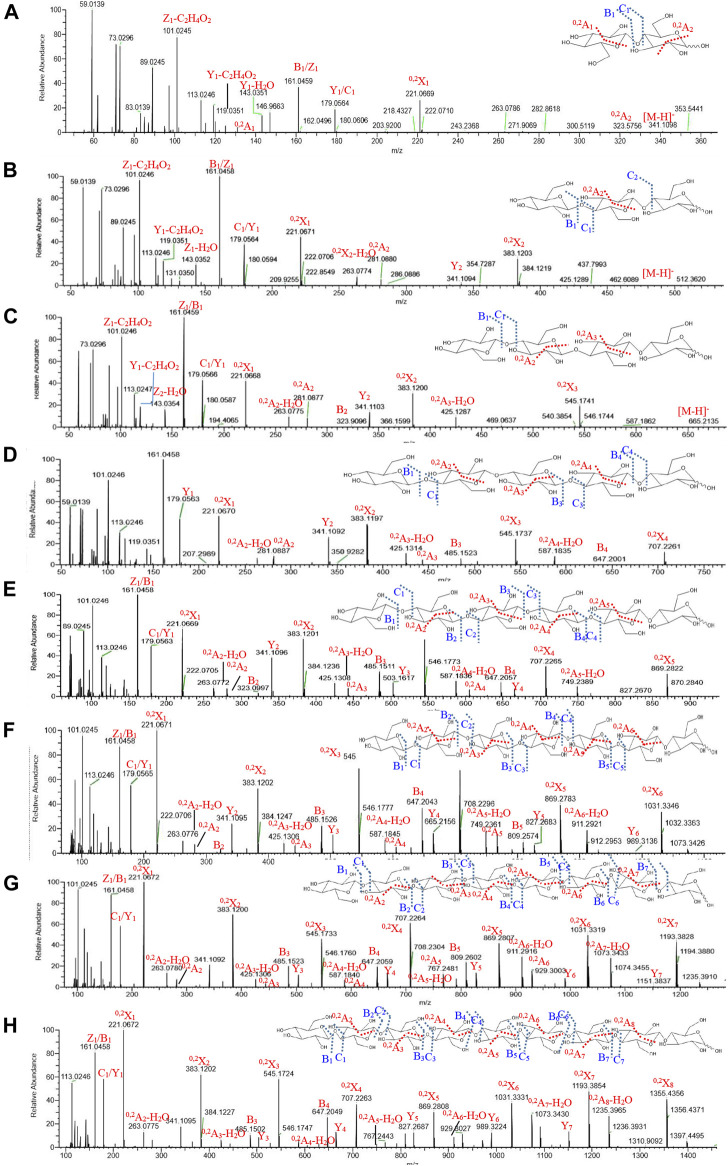
Mass spectra of two to nine sugars from APOS **(A**–**H)**.

The total ion current diagram of APOS is shown in Supplementary Figure S2. In accordance with the definition of Domon and Costello (Ashline et al., 2005), the characteristic ion fragments of different connection types of monosaccharides of APOS were structured. The MS^2^ spectrum of disaccharide (m/z = 342) showed that the reducing end of Glcp residue formed ^0,2^A_2_ cross-ring fragment ions at m/z 323 (Figure 5A). This finding showed that the disaccharide consisted of (1→4)-linked-Glcp residues. The (1→4)-linked nonreducing end residue was fragmented to ^0,2^A_1_ (m/z 120) and ^0,2^X_1_ (m/z 221). All the linear three to nine oligosaccharides were found to produce cross-ring fragment ions in MS^2^ (trisaccharides [m/z = 504]: ^0,2^A_2_ [m/z 281] and ^0,2^A_2_–H_2_O [m/z 263] in Figure 5B; tetrasaccharide [m/z = 666]: ^0,2^A_2_ [m/z 281] and ^0,2^A_2_–H_2_O [m/z 263], ^0,2^A_3_ [m/z 443], and ^0,2^A_3_–H_2_O [m/z 425] in Figure 5C; pentasaccharide [m/z = 827.3]: ^0,2^A_2_ [m/z 281], ^0,2^A_3_ [m/z 443], and ^0,2^A_4_ [m/z 605] in Figure 5D; hexasaccharide [m/z = 990]: ^0,2^A_2_ [m/z 281], ^0,2^A_3_ [m/z 443], ^0,2^A_4_ [m/z 605], and ^0,2^A_5_ [m/z 767] in Figure 5E; heptasaccharide [m/z = 1152]: ^0,2^A_2_ [m/z 281], ^0,2^A_3_ [m/z 443], ^0,2^A_4_ [m/z 605], ^0,2^A_5_ [m/z 767], and ^0,2^A_6_ [m/z 929] in Figure 5F; octasaccharide [m/z = 1314]: ^0,2^A_2_ [m/z 281], ^0,2^A_3_ [m/z 443], ^0,2^A_4_ [m/z 605], ^0,2^A_5_ [m/z 767], ^0,2^A_6_ [m/z 929], and ^0,2^A_7_–H_2_O [m/z 1073] in Figure 5G; nonaose [m/z = 1476]: ^0,2^A_2_ [m/z 281], ^0,2^A_3_ [m/z 443], ^0,2^A_4_ [m/z 605], ^0,2^A_5_ [m/z 767], ^0,2^A_6_ [m/z 929], ^0,2^A_7_–H_2_O [m/z 1073], and ^0,2^A_8_–H_2_O [m/z 1235] in Figure 5H).

The 1→4 glycosidic bond was broken to form B/Z and C/Y ions (disaccharide: B_1_/Y_1_ [m/z 161 and m/z 179], C_1_/Z_1_ [m/z 179 and m/z 161]; trisaccharides: B_1_/Y_2_ [m/z 161 and m/z 341], C_1_/Z_1_ [m/z 179 and m/z 161], B_1_/Y_1_ [m/z 161 and m/z 179]; tetrasaccharide: B_2_/Y_2_ [m/z 323 and m/z 341], C_1_/Z_1_ [m/z 179 and m/z 161], B_1_/Y_1_ [m/z 161 and m/z 179]; pentasaccharide: B_3_/Y_2_ [m/z 485 and m/z 341], B_4_/Y_1_ [m/z 647 and m/z 179], C_1_/Z_1_ [m/z 179 and m/z 161], and B_1_/Y_1_ [m/z 161 and m/z 179]; hexasaccharide: B_2_/Y_4_ [m/z 323 and m/z 665], B_3_/Y_3_ [m/z 485 and m/z 503], B_4_/Y_2_ [m/z 647 and m/z 341], C_1_/Z_1_ [m/z 179 and m/z 161], and B_1_/Y_1_ [m/z 161 and m/z 179]; heptasaccharide: B_1_/Y_6_ [m/z 161 and m/z 989], B_2_/Y_5_ [m/z 323 and m/z 827], B_3_/Y_4_ [m/z 485 and m/z 665], B_4_/Y_3_ [m/z 647 and m/z 503], B_5_/Y_2_ [m/z 809 and m/z 341], B_6_/Y_1_ [m/z 1071 and m/z 179], C_1_/Z_1_ [m/z 179 and m/z 161], and B_1_/Y_1_ [m/z 161 and m/z 179]; octasaccharide: B_1_/Y_7_ [m/z 161 and m/z 1151], B_2_Y_6_ [m/z 323 and m/z 989], B_3_/Y_5_ [m/z 485 and m/z 827], B_4_/Y_4_ [m/z 647 and m/z 665], B_5_/Y_3_ [m/z 809 and m/z 503], B_6_/Y_2_ [m/z 1071 and m/z 241], B_7_/Y_1_ [m/z 1133 and m/z 179], C_1_/Z_1_ [m/z 179 and m/z 161], and B_1_/Y_1_ [m/z 161 and m/z 179]).

**FIGURE 6:**
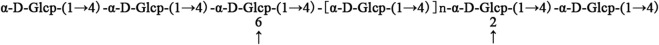
Possible repeating unit of APOS.

The mass spectrum data of the degree of polymerization of two to nine sugars in APOS are shown in Table 4. The saccharides after 10 sugars were not displayed in the MS^2^. The reasons may be that the unstable sugar chains were broken into smaller fragments in the primary mass spectrum, and the corresponding secondary spectrum could not be extracted in accordance with its molecular ions. Combined with methylation analysis and nuclear magnetic analysis, UPLC-ESI-QTOF-MS could further verify that the main chain of the sugar component in APOS was 1,4-linked glucan. The putative structure of the repeating unit of APOS was hypothesized and shown in Figure 6.

**TABLE 4:** Mass spectrometric data of two to nine sugars in APOS.

DP	t_R_/min	Molecular formula	[M-H]^−^, m/z	Mass fragment, m/z
2	1.889	C_12_H_22_O_11_	341	101, 113, 119, 120, 143, 161, 179, 221, 323
3	2.400	C_18_H_32_O_16_	503	101, 113, 119, 143, 161, 179, 221, 263, 281, 341, 383
4	3.125	C_24_H_42_O_21_	665	101, 113, 119, 143, 161, 179, 221, 263, 281, 323, 341, 383, 425, 545
5	4.068	C_30_H_52_O_26_	827	101, 113, 119, 143, 161, 179, 221, 263, 281, 341, 383, 425, 443, 545, 587, 707
6	5.008	C_36_H_62_O_31_	989	101, 113, 119, 143, 161, 179, 221, 263, 281, 341, 383, 425, 443, 503, 545, 587, 605, 665, 707, 749, 827, 869
7	5.986	C_42_H_72_O_36_	1151	101, 113, 119, 143, 161, 179, 221, 263, 281, 341, 383, 425, 443, 503, 545, 587, 605, 665, 707, 749, 827, 869, 911, 989, 1031
8	7.019	C_48_H_82_O_41_	1313	101, 161, 221, 263, 281, 341, 383, 425, 485, 503, 545, 587, 647, 707, 767, 809, 827, 869, 911, 929, 989, 1031, 1073, 1193
9	7.907	C_54_H_92_O_46_	1475	113, 161, 179, 221, 263, 341, 383, 485, 545, 647, 707, 827, 869, 929, 989, 1031, 1073, 1193, 1235, 1355

### Comparison of Non-specific Immune Activity of P1–P4

In non-specific immunity, macrophages can kill pathogens through phagocytosis in the body. Natural killer (NK) cells are important targets for polysaccharide-mediated immune regulation. They can directly identify and kill pathogens, and they have the function of killing tumor cells. This study explored the effects of APS-Ⅱ enzymatic components with different degrees of polymerization on macrophages and NK cells, and their effects on the body’s non-specific immune function were determined (Wang et al., 2020).

**FIGURE 7:**
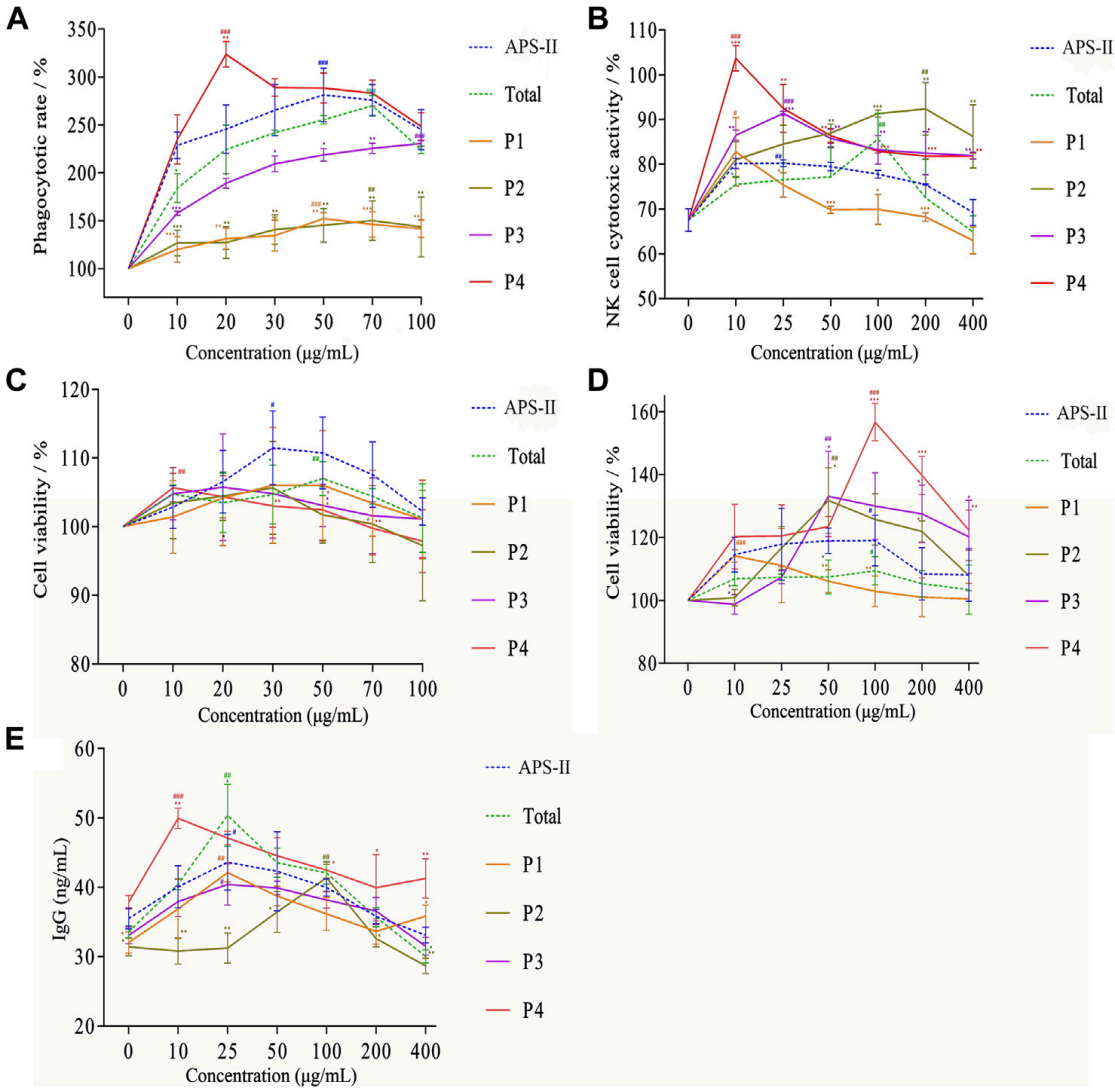
Effects of different saccharide components on phagocytic activity **(A)**, killing activity of mouse spleen NK cells **(B)**, proliferation of B lymphocytes **(C)**, proliferation of T lymphocytes **(D)**, and IgG secretion of splenic lymphocytes **(E)** (*n* = 6, *p < 0.05, **p < 0.01, ***p < 0.001 versus APS-II; ^#^ *p* < 0.05, ^##^ *p* < 0.01, ^###^ *p* < 0.001 versus blank control group).

Raw264.7 macrophages are the major component in the innate immune system. They function as one of the earliest lines of defense against invading pathogens (Fernando et al., 2014). Neutral red is a large-molecule fluorescent reagent. Given its large molecular weight, it can only be taken into cells through phagocytosis (Liu QM. et al., 2017). In the present study, the neutral red method was used in detecting the effects of different saccharide components on the phagocytic activity of Raw264.7 cells (Figure 7A). Compared with the blank group, the phagocytic activity of Raw264.7 cells with different saccharide components at the optimal dose was statistically significant (*p* < 0.05). At low concentrations, the phagocytic activity of Raw264.7 cells increased with increasing concentrations of APS-Ⅱ, APOS, and P4. The optimal concentrations of APS-Ⅱ, APOS, and P4 were 50, 70, and 20 μg/ml, respectively. The enhancement effect gradually decreased as the concentration was further increased, and the dose–effect curve was “bell-type.” The polysaccharide immune function was regulated in two manners, and an optimal dose was present. At different concentrations, the order of different saccharide components on the phagocytic activity of Raw264.7 cell was P4 > APS-II ≈ APOS > P3 > P1 ≈ P2, which may correlate with the structural feature of the polysaccharides. APS-II and APOS had similar effects on the phagocytic activity of Raw 264.7, indicating that APS-II could be degraded into small fragments to exert their phagocytic activity. The P4 component had the greatest effect on the phagocytic activity of Raw264.7 cells, and this finding may be related to the structural characteristics of P4.

Natural killer cells are also a type of lymphocytes that could non-specifically kill tumor cells and virus-infected cells without pre-sensitization. The effects of different saccharide components on the killing activities of NK cells are shown in Figure 7B. Different saccharide components could promote the killing activity of mouse spleen NK cells, and the dose–effect curve was “bell-type.” P1, P2, P3, P4, APOS, and APS-II at doses of 10, 200, 25, 10, 100, and 25 μg/ml had the strongest killing activities against NK cells, respectively. Compared with the blank group, the NK cell killing activity of saccharide components with different degrees of polymerization was significantly different at the optimal dose (*p* < 0.05). The order of the different saccharide components to promote the killing activity of NK cells was P4 > P3 ≈ P2 > APS-II ≈ APOS > P1. APS-II and APOS had similar effects in enhancing the killing activity of NK cells, indicating that APS-II could be degraded into small fragments to promote the killing activity of NK cells. The P4 component exhibited the greatest effect on the killing activity of NK cells.

### Comparison of Specific Immunological Activity of P1–P4

Lymphocytes are an important part of the body’s immune response function. Among them, B and T lymphocytes have specificity, and they share the body’s humoral and cellular immune functions during the immune response process. LPS and ConA are mouse B cell-specific mitogen and T cell-specific mitogen, respectively, and they could promote cell metabolism. In addition, the level of IgG produced by splenic lymphocytes could reflect the status of humoral immunity. APSs not only could enhance the proliferation ability of lymphocytes but also increase the secretion of cytokines, thereby enhancing the immune function of the body. Therefore, this study explored their effects on the body’s specific immune function by studying the effects of APS-Ⅱ enzymatically hydrolyzed sugar components with different degrees of polymerization on the B and T lymphocytes induced by LPS and ConA and the level of IgG produced by splenic lymphocytes (Yu et al., 2020).

The effect of LPS combined with different saccharide components on the proliferation of B lymphocytes is shown in Figure 7C. P1, P2, and P3 had no obvious effects on the proliferation of B lymphocytes. The optimal concentration of P4 was 10 μg/ml. When the concentration of APOS was 50 μg/ml, the effect on the proliferation of B lymphocytes was the strongest. APS-II had the strongest effect on the proliferation of B lymphocytes at 30 μg/ml. Compared with the blank group, the optimal doses of these three components significantly varied (*p* < 0.05), and all of them significantly promoted the proliferation of B lymphocytes. This result showed that the degradation of APSs into small fragments could exert specific immune effects by enhancing LPS-induced proliferation of B lymphocytes. Similarly, the effect of ConA combined with different saccharide components on the proliferation of T lymphocytes is shown in Figure 7D. The order of these saccharides on the proliferation activity of T lymphocytes was as follows: P4 > P3 ≈ P2 > P1 > APOS ≈ APS-II. P4 had the strongest effect on the proliferation of T lymphocytes. APS-II and APOS had similar effects in enhancing the proliferation of lymphocytes, indicating that APS-II could be degraded into small fragments to induce lymphocyte proliferation and exerts specific immune effects.

The effect of different saccharide components in conjunction with LPS on the secretion of IgG from mouse splenic lymphocytes is shown in Figure 7E. Compared with the blank group, the optimal doses of different components were statistically different (*p* < 0.05). They all significantly promoted the secretion of IgG from mouse spleen lymphocytes. The order of these saccharides with regard to capability to promote IgG secretion in splenic lymphocytes was as follows: P4 > APOS ≈ APS-II > P1 ≈ P3 > P2. This finding showed that APS-II could be degraded into small molecular fragments, and it exerts specific immune effects by promoting the secretion of IgG from splenic lymphocytes. Among them, the strongest activity was sugar with a degree of polymerization of 10–18.

In summary, APS-II and its enzymolysis products could enhance the phagocytic function of macrophages and the killing activity of NK cells, thereby promoting non-specific immune function. In addition, it could cooperate with ConA to promote the proliferation of T lymphocytes and with LPS to promote the proliferation of B lymphocytes and secrete cytokine IgG, thereby enhancing the specific immune function. Different saccharide components all showed certain activity. Among them, the P4 component exhibited the strongest activity and was higher than the immunologically active polysaccharide APS-II. Combined with the results of structural analysis, the proportion of →2,4)-Glcp-(1→ (25.34%) and →4,6)-Glcp-(1→ (34.54%) in the sugar chain of P4 was the largest among the components. Arabinose, galactose, and glucose residues were attached to the C2 and C6 branches. The immune activity of P4 was closely related to its structure.

### Correlation Relationship Analysis Between Structure and Activity of Enzymolysis Components From APS-Ⅱ

The chemical structure of polysaccharides is the basis for its biological activity. However, due to the complexity of the structure of polysaccharides and the limitations of research methods, the research of polysaccharides has always lagged behind proteins and nucleic acids. Clarifying the structure–activity relationship of polysaccharides is the focus of glycochemistry and glycobiology. However, the various activities of polysaccharides are closely related to their physicochemical characteristics. Minor changes in the ratio and sequence of monosaccharides, molecular weight, type of glycosidic linkage, and degree of branching could notably affect the biological characteristics of polysaccharides (Liu Y. et al., 2021; Chen et al., 2021; Luan et al., 2021).

Studies have shown that the optimal activity of polysaccharides depends on their relative molecular mass. The relative molecular mass needs to be within a suitable range to exert the best activity. The larger the relative molecular mass is, the larger the volume of the polysaccharide molecule, and its transmembrane resistance increases accordingly, which is not conducive to absorption and utilization, thereby affecting its immune activity. However, if the relative molecular mass is too low, the polysaccharide cannot form an active structure, thereby reducing its activity (Gong et al., 2017). In the earlier stage, the immune activity of three different Mw components in APSs was compared and the results showed that APS-Ⅲ is mainly monosaccharide or disaccharide, which has no significant effect on enhancing the immune function of the body. However, APS-II with a molecular weight of 10 kDa had a better immunomodulatory effect than APS-I (molecular weight, >2,000 kDa). Although APS-I had certain biological activity, its relatively large molecular weight, poor solubility, and low bioavailability limit its efficacy (Li et al., 2020a). Therefore, the immunological activity of APSs is closely related to its molecular weight. In the present study, P4 had the strongest activity in specific and non-specific immunity compared to the other fractions, which indicated that the molecular weight (1639–2935) of P4 may be suitable for development as drugs. In fact, low molecular weight heparin drugs have similar characteristics of molecular weight distribution.

In addition, polysaccharides are usually composed of various monosaccharides connected by glycosidic bonds. Glycosidic bonds play an important role in the activity of polysaccharides. One of the key points is the local conformation of the polysaccharide molecular chain, which is determined by the glycosidic bonds between sugar groups. Li et al. (2009) pointed out that α-(1→4)-D-glucan in the structure of APS may be an important factor affecting its immunomodulatory activity. The existence of this structure could significantly enhance the immunity of rats with gastric cancer. This study provides a basis for the use of APSs in the treatment of gastric cancer. Wei et al. (2018) reported that the polysaccharide extracted from the culture of *Rhizopus nigricans* is a α-(1→4)-glucose and observed a branch at the C-6 position, which improved the immune function of tumor-bearing mice and significantly inhibited the growth of transplanted tumors. The degree of branching is closely related to the biological activity of polysaccharides. If the degree of branching is too large or too small, the biological activity of the polysaccharide could not reach the ideal state (Bohn and Be Miller., 1995). After debranching, the activity of polysaccharides decreases significantly (Ren et al., 2012). Bohn and Be Miller. (1995) compared the degree of branching of dozens of glucans with their antitumor activity and believed that β-1,3-D-polysaccharides with a degree of branching between 0.20 and 0.33 have higher biological activity. In the present study, the branched chains at positions C2 (25.34%) and C6 (34.54%) on the sugar chain of the α-(1→4)-glucan of the P4 component accounted for a higher proportion than those in the other three degraded components, and more arabinose, galactose, and glucose residues were attached to the branch chain. The P4 component showed the strongest immunological activity, indicating that the activity of the enzymolysis components from APS-Ⅱ is closely related to the glycosidic bond connection mode of each component and its branching degree.

In summary, in this study, P4 had a higher degree of polymerization and a larger molecular weight than the other three degraded components. In the α-D-1,4-linked glucan structure of the main chain, the branches at the C-2 and C-6 positions accounted for a relatively high proportion, and different monosaccharide residues were found, which may be the key structure for its immune regulation. This finding was consistent with the conclusions of existing studies (Yan et al., 2011; Ferreira et al., 2015).

## Conclusion

This study adopted a “bottom-up” research strategy. APS-II endo α-1,4-glucanase digestion components with different degrees of polymerization were obtained through polyacrylamide gel chromatography. The structures of P1, P2, and P3 were linear 1,4-linked glucans. However, the number of glucose branch chains in P3 increased relative to that in P1 or P2. In P4, the main connection methods of sugar chain were 1→2,4-Glcp (25.34%) and 1→4,6-Glcp (34.54%). Glucose with a high degree of branching was the main component in the sugar chain.

The immune activity of mixtures of saccharides with different degrees of polymerization was compared. APS-II and APOS had similar effects in the development of non-specific and specific immunity, indicating that APS-II could be degraded into various small molecular fragments to play an immune-promoting effect. Among the saccharide components with different degrees of polymerization, P4 (10–18 sugars) showed the strongest activity in terms of enhancing the phagocytic activity of macrophages, enhancing the killing activity of NK cells, promoting the proliferation of lymphocytes, and promoting the secretion of cytokine IgG from lymphocytes. Thus, P4 with repeating α-D-1,4-linked glucans backbone with C2 (25.34%) and C6 (25.34%) branches has the strongest immunological activities, providing a theoretical basis for the study of structure–activity relationship of APS-Ⅱ. This study lays the foundation for the further structure–activity relationship of APSs and provides guidance for the quality control of APSs and the development of new drugs.

## Data Availability

The original contributions presented in the study are included in the article/Supplementary Material, further inquiries can be directed to the corresponding author.
