# Supplementary material for: Relationship Between the Structure and Immune Activity of Components From the Active Polysaccharides APS-II of Astragali Radix by Enzymolysis of Endo α-1,4-Glucanase
Source: Front Pharmacol. 2022 Feb 25;13:839635. doi: 10.3389/fphar.2022.839635 (PMC8913491; doi:10.3389/fphar.2022.839635)
Supplement: Supplementary file 1 [file DataSheet1.docx]

**Supplementary data**

A

B

C

D

E

Figure S1. GC–MS total ion chromatogram of P1(A), P2 (B), P3(C), P4(D), and APOS (E)

Figure S2. UPLC-ESI-QTOF-MS total ion chromatogram of APOS
